# Phyllosphere bacterial assembly in citrus crop under conventional and ecological management

**DOI:** 10.7717/peerj.9152

**Published:** 2020-06-02

**Authors:** Carolinne R. Carvalho, Armando CF Dias, Sérgio K. Homma, Elke JBN Cardoso

**Affiliations:** 1 Department of Soil Science, College of Agriculture “Luiz de Queiroz”, University of São Paulo, Piracicaba, São Paulo, Brazil; 2 Mokiti Okada Research Center, Ipeúna, São Paulo, Brazil

**Keywords:** Phyllosphere, Agricultural management, Agroecology, Foliar copper, New generation sequencing, XRF

## Abstract

Divergences between agricultural management can result in different types of biological interactions between plants and microorganisms, which may affect food quality and productivity. Conventional practices are well-established in the agroindustry as very efficient and lucrative; however, the increasing demand for sustainable alternatives has turned attention towards agroecological approaches. Here we intend to explore microbial dynamics according to the agricultural management used, based on the composition and structure of these bacterial communities on the most environmentally exposed habitat, the phyllosphere. Leaf samples were collected from a Citrus crop (cultivated Orange) in Mogi-Guaçu (SP, Brazil), where either conventional or ecological management systems were properly applied in two different areas. NGS sequencing analysis and quantitative PCR allowed us to comprehend the phyllosphere behavior and µ-XRF (micro X-ray fluorescence) could provide an insight on agrochemical persistence on foliar tissues. Our results demonstrate that there is considerable variation in the phyllosphere community due to the management practices used in the citrus orchard, and it was possible to quantify most of this variation. Equally, high copper concentrations may have influenced bacterial abundance, having a relevant impact on the differences observed. Moreover, we highlight the intricate relationship microorganisms have with crop production, and presumably with crop yield as well.

## Introduction

The phyllosphere is composed of the above-ground plant surface that is inhabited by microorganisms, being mostly represented by foliar tissue (Bodenhausen et al., 2014). Since the leaves are very exposed to environmental variations, several factors may be able to modulate the microbial assemblage (Dias et al., 2012; Redford et al., 2010; Truchado et al., 2017). Geographic location, temporal variation, plant genotype and stage of development have been demonstrated to strongly modulate the microbial diversity in the phyllosphere (Finkel et al., 2011; Lambais, Lucheta & Crowley, 2014; Truchado et al., 2017; Vorholt, 2012). In this context, we questioned how much agricultural management may influence bacterial communities in the phyllosphere, hypothesizing that different implemented practices can lead to changes in the structure and abundance of microorganisms.

Conventional agriculture, for long periods of time, has been the choice of management for crop cultivation, owing to its highly desired results, as high productivity and agricultural winnings (Mazzoncini et al., 2010). However, these practices often cause severe long-term effects on the environment, changing the microbiome composition and its functional diversity (Geertsema et al., 2016; Krauss, Gallenberger & Steffan-Dewenter, 2011). As an alternative, ecological agriculture has increasing visibility in the agroindustry, defending the use of ecological services, promoted by the ecosystem itself, and presenting greater sustainability (Oelofse et al., 2011; Altieri, 2013; Office of the United Nations High Commissioner for Human Rights (OHCHR), 2017). There are very scarce studies aiming at evaluating agricultural management effects on the bacterial phyllosphere diversity, although this micro-environment could provide information on the consequences of agricultural practices, and plant fitness status as well. In reality, most phyllosphere studies have been directed to forest trees from several biomes (Martins et al., 2012; Ottesen et al., 2016; Pagadala et al., 2015; Perazzolli et al., 2014). We have chosen to evaluate citrus plants because of the great economic and social importance of this crop for Brazil (Brazilian Agricultural Research Corporation (EMBRAPA), 2016). Since there are many knowledge gaps concerning agricultural management effects on the microbiome associated to plants, we formulated our above-mentioned hypothesis on the factors that govern their modifications.

One very relevant practice used in agriculture for disease-control is the application of copper-based products as fungicides and bactericides. Copper application on foliar tissues is commonly used in citrus farming as it combines high efficiency and low toxicity to the plant, which is accepted in both, conventional or agroeocological management systems, although in different concentrations (Mackie et al., 2013; Schutte, Van Zyl & Fourie, 2012). However, copper-based products have a microbial broad-spectrum, which means, that they do not only act on plant pathogens, but also indistinctly on other phyllosphere residents (Martins et al., 2012; Varanda et al., 2016).

In this study, our aims were (i) to characterize the composition of the bacterial communities of the citrus phyllosphere under different management systems, (ii) to identify copper distribution on citrus foliar surfaces, and (iii) to compare the variation of the phyllosphere bacterial communities according to the farming system approaches. We hypothesize that variations in agricultural management will be a significant driver of the phyllosphere bacterial community structure.

## Materials and Methods

### Sampling location

The experimental area is located in the Santo Antônio do Lageado farm (22°08′49,4″S and 47°10′47,6″W), in Mogi Guaçu, São Paulo State, Brazil. A citrus orchard of 4.50 ha, that was planted in 2007 and cultivated under conventional management up to 2011, when this trials was started under supervision of the Mokiti Okada Research Center (CPMO), a non-profit center based in Ipeúna in the state of Sao Paulo. The area was subdivided into two parts, each one under a different agricultural management system, one half being maintained under conventional (CO), and the other half under ecological management (EC). However, the ecologically managed part initially was submitted to a 5-year transition regime, in which the management practices were slowly modified from one system to the other. We initiated this study on the structure of the citrus phyllosphere, when the area under ecological management reached the 5th year of transition. In Table 1 we describe the chemical applications used in both management systems during the sampling period (September and December/2016), according to CPMO definitions.

**Table 1:** Pesticide application chronogram in the studied area. Agricultural pesticides applied (per hectare) during the sampling period of three months of crop field evaluation, according to Brazilian Agricultural laws and MOA parameters.

Type of product	Active compound (%)	Dry (Sep/2016)		Wet (Dec/2016)	
		CO	EC	CO	EC
Fungicide	Copper oxychloride (84 %)	7.2 Kg	3.6 Kg	1.8 Kg	–
	Copper hydroxide (65.6%)	–	–	2 Kg	2 Kg
	Estrobirulin (25%)	–	–	0.3 L	–
Insecticide	Pyrethroids (5%) + anthranilamide (10%)	0.2 L	–	–	–
	Neonicotinoids (70%)	–	–	0.24 Kg	–
Acaricide	Abamectin (1.8%)	1L	–	1L	–
Leaf fertilizer	Fertilizers + surfactants	1L	–	1L	1L
Others	Vegetal oil	1L	–	–	–

**Note:**

– Without any application.

### Leaf sampling and experimental setup of DNA extraction

Healthy-looking leaves were sampled from individual citrus trees (*Citrus sinensis* L. Osbeck) using a hand shear to cut off the branches, manipulating them with sterile gloves to avoid contamination, and disposing them directly into plastic bags (Lambais, Lucheta & Crowley, 2014). We sampled about 40 leaves from each 20 trees in each area for molecular analysis, and these leaves were later kept at −20 °C for a short-time analysis. The sampling periods were in September (end of the dry season) and in December 2016 (rainy season). To get the superficial bacteria, the leaves were placed in 200 mL erlenmeyer flasks, containing 50 mL of 0.9% saline solution and were shaken at 150 rpm for 1 h 30 min to wash off the bacteria. Afterwards, the bacterial suspension was sonicated for 20 min at 22.5 kHz with an ultrasonic cell disruptor in order to release all phyllosphere microorganisms, while their total DNA was extracted using the phenol/chloroform method, according to Rigonato et al. (2012). For cell lysis, glass beads (0.1 mm) were added, in order to provoke mechanical disruption in a bead beater device (BioSpec, Bartlesville, OK, USA) for about 45 s. The pellet obtained from the resulting bacterial suspension was suspended in 500 µL of TE (Tris-EDTA buffer), and amended with 10% sodium dodecyl sulfate (SDS). The DNA was re-suspended using 50 µl of mili Q purified water and then preserved at −20 °C for future procedures.

### Sequence-based analysis of bacterial communities

In order to access the phyllosphere bacterial community structure, the total DNA was extracted from the previously prepared bacterial suspension and was purified. From initial total DNA samples, we randomly selected triplicates from both CO and EC areas during the dry weather period for the paired-end 16S rRNA sequencing. A bacterial 16S rRNA gene library was prepared according to the sequencing library preparation protocol (Illumina Inc., San Diego, CA, USA) using a set of primers with the forward 515F 5′-GTGCCAGCMGCCGCGGTAA and reverse 806R 5′-GGACTACHVHHHTWTCTAAT searching specifically for the V3–V4 region (Caporaso et al., 2011). Resulted amplicons were sequenced using the MiSeq Reagent kit V3 (600 cycles) using Illumina MiSeq^®^ System platform with 2 × 250 bp paired-end sequencing. The samples were processed and treated with Wizard^®^ SV Gel and with the PCR Clean-Up System Kit for purification and then pooled in equal accurate concentrations. Sequencing happened at the Multiusers Laboratory of Applied Functional Genomics for Agriculture and Agro-energy at ESALQ, University of São Paulo, Brazil.

### Bioinformatics data processing

We analyzed the raw sequence data with QIIME^™^ 1.9.1 pipelines to merge paired-end sequences to single scaffolds, and attributed taxonomic identity to the obtained operational taxonomic units (OTUs), also determining the phylogenetic structure and bacterial diversity (Caporaso et al., 2010). Low quality sequences (quality score 20) were removed and, using the function “multiple_split_libraries_fasta.py”, all samples were compiled in one fasta file. Sumaclust algorithm was used for clustering the sequences with high accuracy, respecting their stability and heritability (Jackson et al., 2016). Then, we binned the remaining sequences into OTUs at 97% sequence similarity cut-off, crossing reference databases using Sumaclust (“pick_otus.py”) (Kopylova et al., 2014). Taxonomy identity of each OTU was determined using Uclust algorithm and SILVA database (Yilmaz et al., 2014), a reference aligned as implemented in QIIME (“parallel_assign_taxonomy.py”). Due to the similarity of 16S ribosomal regions between bacteria with chloroplasts and mitochondria, it was necessary to filter the sequences to avoid mistaken assumptions (“filter_taxa_from_otu_table.py”). A total count of 258,256 16S rRNA gene sequences was obtained from six phyllosphere samples, and the raw OTU table was rarefied to 12,300 sequences with good quality. Alpha and beta diversity metrics were analyzed through phylogenetic distance, the weighted Unifrac distance matrix (“core_diversity_analysis.py”), and the Chao and Shannon index (Caporaso et al., 2010). The similarity of the bacterial diversity between the management areas was analyzed by a principal coordinate analysis (PCoA), similarity percentage (SIMPER) and PERMANOVA calculations were conducted using the PAST (version 2.17c, 2013) software (Hammer, Harper & Ryan, 2001), based on Euclidian and Bray-Curtis dissimilarity, respectively.

### Quantitative PCR

Quantitative qPCR analysis was performed on total samples from each area using StepOne^™^ Real-Time PCR System (Applied Biosystems^®^, Foster City, CA, USA), using primers P1 (5′-CCTACGGGAGCGAGCAG-3′) and P2 (5′-ATTACCGCGGCTGCTTGG-3′) specific for the 16 rRNA region of bacteria. Here, initial total samples were analyzed, including samples for previous community analysis in order to get better inference about bacterial abundance. SYBR^®^ Select Master Mix (Muyzer, De Waal & Uitierlinden, 1993). Each reaction was performed in a volume of 20 µl containing 10 µl SYBR, 0.5 µl of each primer (10 mM), 1 µl DNA and 8.0 µl miliQ water. The reaction followed the sequence of three min initial denaturation at 95 °C, 35 cycles of 30 s at 94 °C, 30 s at 55 °C and 30 s at 72 °C, followed by a melting curve analysis (Lopes, De Cássia & Andreote, 2016; Muyzer, De Waal & Uitierlinden, 1993). Fluorescence was detected during the extension step of each cycle, and the *C*_T_ values plotted against log to obtain the gene copy number. The concentration of the total DNA (ng/µl) was determined using Qubit fluorometry (Invitrogen^™^, Carlsbad, CA, USA). Bacterial abundance was then calculated and expressed in gene copy number per ng of DNA. Both measurements were done in triplicates. Quantitative data obtained for both CO and EC areas were analyzed by ANOVA and Tukey’s test at 5% using R 3.4.1 software.

### Mapping Cu accumulation spots on foliar tissue

Leaf sampling for copper mapping analysis followed the MOA’s schedule for copper-based product applications in the experimental area during the dry season. The first sampling occurred at the first day right after copper application (*t* = 0), and then 15 days later, in order to observe temporal changes in Cu accumulation (*t* = 1). Leaves were taken from the same sampled trees for molecular analysis, although imaging was done in triplicates for each area at each period. We used 2–3 leaves per imaging and applied the technique in duplicates for each treatment each time evaluated. Sampled leaves were dried in an incubator at 50 °C for 72 h to get a low and uniform moisture content. The microanalysis was carried out using a micro-X-ray fluorescence spectroscopy (µ-XRF) system (Orbis PC; EDAX, Mahwah, NJ, USA), according to proper specification (Duran et al., 2017) at the Laboratory for Nuclear Instrumentation, Center of Nuclear Energy in Agriculture, University of São Paulo, Brazil. Micro-XRF mapping of the distribution of Cu on the leaf surfaces was performed with an incident energy at 30 KeV. The storage ring current during data acquisition was 300 µA (continuous). The beam was focused to one mM during two seconds per point. The chemical Cu maps were generated using the software EDAX Spectral Processing Utilities, in which the images were constructed with X-ray-emitted photons irradiating a greater or smaller number of spots on the leaf. Due to the varying thickness of the foliar tissue, Compton’s matrix correction was addressed to obtain accurate imaging of biological tissues (De Carvalho et al., 2018). The provided matrices to compose the graphic maps were analyzed using OriginPro^®^ 2017 software.

## Results

### Characterization of phyllosphere communities with illumina MiSeq

**Figure 1:**
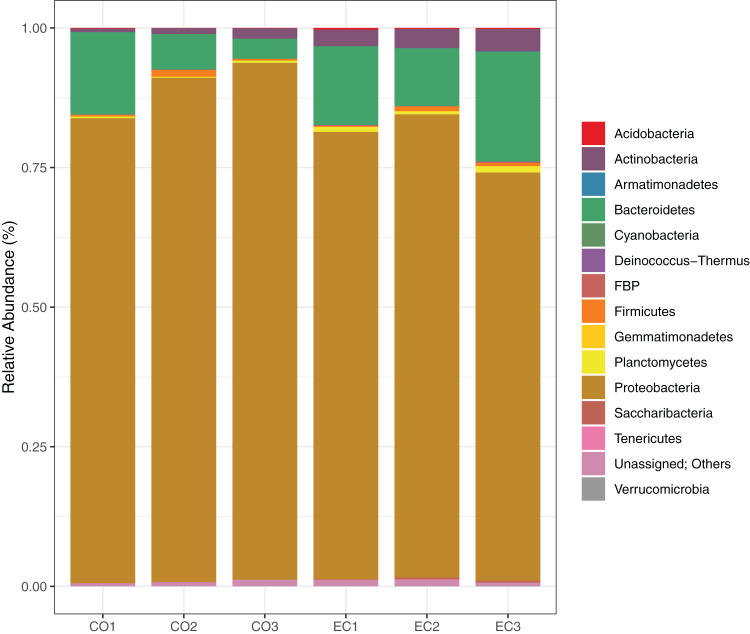
Bacterial distribution in the phyllosphere at the phyllum level. The graphic shows the results for 16S rRNA sequencing using Illumina MiSeq plataform, demonstrating bacterial taxonomy and relative abundance for triplicate samples from conventional and ecological areas, respectively.

We show the results of the sequencing analysis according to phylum distribution level in Fig. 1. A total amount of 1,921 OTUs (with 97% of identity) were obtained from both orchard areas, with a mean of 280 OTUs from the conventional (CO) and 359 from the ecological (EC) site (Table 2). The bacterial OTUs were assigned to 14 different phyla using SILVA database, in which Proteobacteria (83.7%), Bacteroidetes (11.4%), Actinobacteria (2.3%) and Firmicutes (0.6%) dominated the phyllosphere in both, CO and EC treatments. At family level, we provided SIMPER analysis (Bray-Curtis index) to determine which families mostly differ between areas, thus contributing to management dissimilarities in microbial richness. The results showed that the dissimilarity contributions from bacterial families were about 21.82%, and the ones which most contributed in the CO treatment were *Methylobacteriaceae*, *Moraxellaceae* and *Enterobacteriaceae*, while *Cytophagaceae* and *Nocardiaceae* were dominant in EC, together with several other groups, as shown in Table 3.

**Table 2:** Diversity measures based on sequencing data. Number of sequences, sample-specific number of OTUs, species richness estimates Chao1 and diversity indices calculated for each sample.

Sample ID	Number of sequences*	Number of OTUs	Phylogenetic diversity whole tree	Richness estimation (Chao1 97%)	Shannon index (H)
CO1	12,339	205	20.011	451.5	1.395
CO2	37,252	289	28.194	840.6	1.641
CO3	46,658	348	33.645	644.2	1.892
EC1	51,319	437	37.217	816.9	2.786
EC2	52,639	368	32.060	666.3	2.464
EC3	58,049	274	27.431	689	2.056

**Note:**

* Non-rarefied values.

**Table 3:** SIMPER analysis. SIMPER analysis results displaying the top ten OTUs accounting for the dissimilarity between managements.

Family level	Contribution (%)^1^	Mean abundance^2^	
		Conventional	Ecological
Methylobacteriaceae	40.71	0.731	0.556
Cytophagaceae	17.33	0.0754	0.139
Nocardiaceae	8.954	0.0694	0.108
Oxalobacteraceae	4.479	0.0056	0.0249
Rhizobiales; 1174-901-12	4.202	0.0201	0.0381
Microbacteriaceae	3.967	0.00847	0.0255
Moraxellaceae	3.144	0.0147	0.00199
Enterobacteriaceae	2.689	0.0201	0.0119
Acetobacteriaceae	2.652	0.0069	0.0183
Sphingobacteriaceae	1.355	0.000202	0.00602

**Notes:**

1 Contribution of OTUs to the overall dissimilarity between groups.

2 Average abundance of OTUs in each groups.

**Figure 2:**
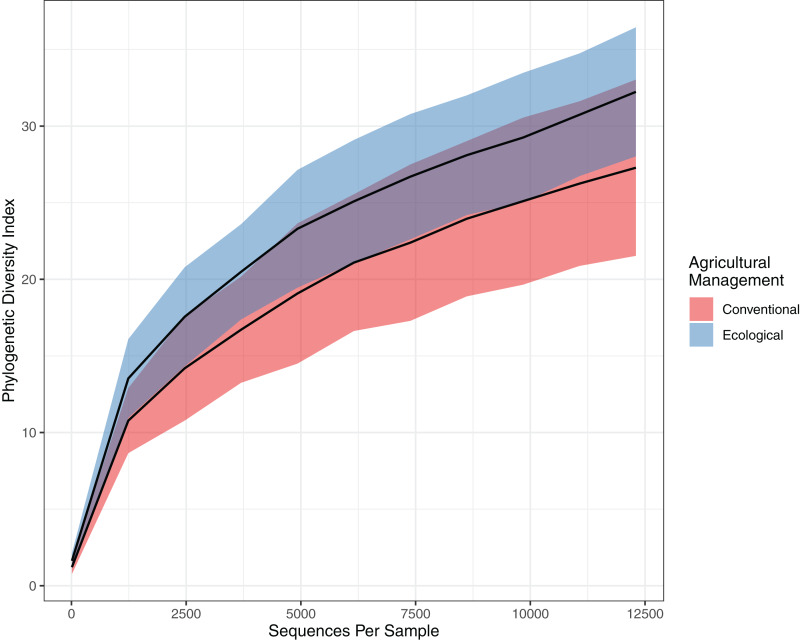
Comparison of alpha diversity for bacterial community. Rarefaction curve representing the alpha diversity of the bacterial community sctructure of the citrus phyllosphere under ecological and conventional management.

This tendency towards dissimilarity in bacterial diversity was explored deeper by using other statistical approaches. To estimate α-diversity, we drew a rarefaction curve, based on phylogenetic diversity, which compares areas according to phylogenetic data obtained for each sample. Both rarefaction curves tended to approach a plateau, indicating an acceptable data amount of sequenced reads. Indeed, when comparing the rarefaction curves, the phylogenetic diversity from EC (32.236 ± 3.99) is continuously higher than the CO values (27.283 ± 5.60), presenting a slightly higher richness and higher abundance (Fig. 2). However, the Chao estimator for species richness confirms that microbial richness does not statistically differ between conventional and ecological areas (*p*-value > 0.05) (Table 2). On the other hand, in terms of diversity, Shannon’s index demonstrates higher diversity from ecological samples, in which Krukal-Walis analysis reinforce significant differences with *p*-values of <0.05.

**Figure 3:**
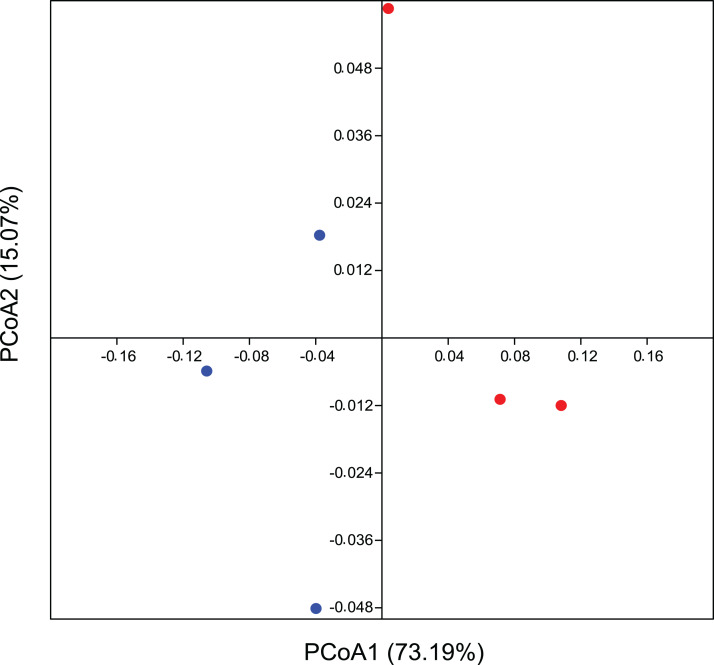
Beta diversity for bacterial community using multivariate statistics. Coordinate analysis (PCoA) demonstrating bacterial community assemblage in conventional agriculture (red) and ecological (blue), based on the weighted Unifrac method. Data is based on 16 rRNA gene sequences. The eigenvalues displayed on the diagram axes refer to the percentage variation of the respective axis.

In addition, the PCoA analysis based on the weighted Unifrac method also identified differences between the areas. The PCoA demonstrates a β-diversity, in which the CO samples formed separate clusters from the others originating from the EC treatment, with a spatial variability of data explained in 73.2% by axis 1 (Fig. 3). Considering a *p*-value > 0.09, we ran a PERMANOVA on Bray-Curtis distance analysis for 999 permutations and we understand as quite significative these differences between groups. These results corroborate our hypothesis of total bacterial diversity differing between management systems.

### Temporal and treatment distinction of bacterial abundance in the phyllosphere

As a complementary analysis of microbial community changes, we included an evaluation of the bacterial abundance of both areas during two different climatic periods (dry and rainy). The *q*PCR shows the number of copies per ng of DNA (Table 4). There is a significant difference between both evaluated areas during the dry period (the same sampling period for the NGS analyses), when the EC values showed a significantly greater abundance than the CO (*p* = 0.0009). However, when analyzing the results from the rainy period, there was an expressive quantitative loss in bacterial abundance from EC (*p* = 0.69). Conversely, we did not identify any significant difference between the two temporal measurements for CO abundance.

**Table 4:** qPCR relative abundance results. Total bacterial abundance in the phyllosphere under conventional and ecological management during dry and wet weather. Number of DNA copies per nanogram of DNA.

Agricultural management	Mean abundance	±SD	Mean abundance	±SD
	Dry weather		Wet weather	
CO	4.79	0.88	2.76	0,48
EC	13.39*	5.63	1.95**	0,50

**Notes:**

* Significant difference when comparing between managements during dry weather (*p* < 0.05).

** Significant difference when comparing EC along a time period (*p* < 0.05).

### Spatial distribution of Cu on citrus leaf tissue

**Figure 4:**
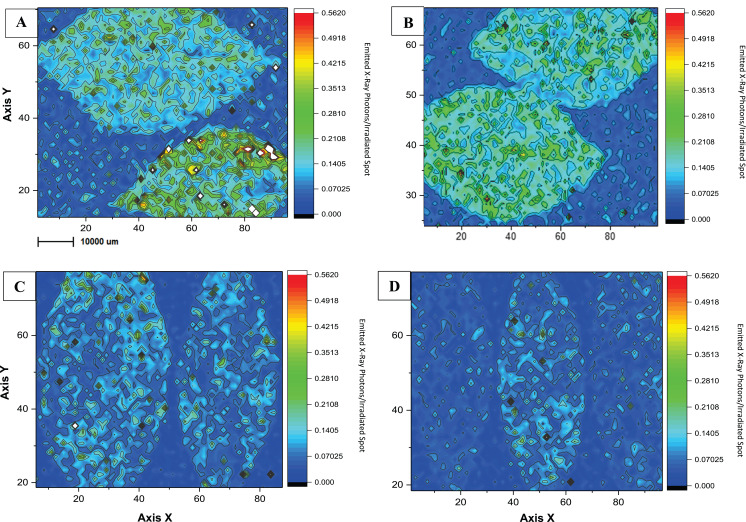
Microchemical maps obtained by micro-XRF for Cu in the surface of *Citrus* sp. leaves. Microchemical maps obtained by micro-XRF for Cu in the surface of *Citrus* sp. leaves conventional (A and B) and ecological management (C and D) on copper-based products application day (on the left) and 15 days-after (on the right). The Rh K-alpha Compton peak was used for correcting the maps.

As an initial investigation of copper distribution on foliar tissues, we used a procedure of mapping and analyzing copper traces on citrus leaf surfaces (Fig. 4). The imaging gave us an interesting lead to find out how differences in agricultural management can affect chemical plant liability, since the application of copper-based products differed in applied concentrations and adjuvants between the two experimental farming areas. To the best of our knowledge, this is the first study to present an evaluation of µ-XRF in citrus tissues in order to compare agricultural treatments. XRF is a non-destructive technique, comprehending a few steps of sample preparation, and its use has increased for diagnoses in plant nutrition and chemical accumulation (De Carvalho et al., 2018). Figure 4 shows the local and temporal comparison of the copper mapping of leaves from the conventional (CO) and the ecological (EC) areas during the dry season to avoid the possible leaf wash-off by the rain. When comparing the managements maps, it is possible to identify greater amounts of Cu on samples from the CO treatment, and this difference remained even after a 15 day-period of pesticide application. Supplemental Material presents map duplicates, which demonstrate to follow the same observations for qualitative measures (Fig. S1). There was no significant visible difference of Cu distribution between both climatic sampling periods, probably due, at least partially, to the co-application of adjuvants such as vegetal oil (Fernández, Sotiropoulos & Brown, 2015; Srivastava, 2012), which help to fix the metal on the organic material. No foliar defects due to excess of Cu were identified in our experimental area.

## Discussion

In this study, we explored the citrus phyllosphere with regard to different management approaches in order to characterize their bacterial composition, as well as their structure over time and according to agricultural management. Firstly, we investigated the composition of these phyllosphere communities using Illumina-based 16S rRNA gene sequencing. We focused on samples from the dry season, in order to avoid washing-off of the leaf surfaces, which could have interfered in the diversity analysis. The most prevalent groups were *Proteobacteria, Bacteroidetes, Actinobacteria* and *Firmicutes*, regardless of the treatment applied (Fig. 1). These groups are commonly cited in the literature as ‘core’ residents of the phyllosphere region in several plant species (Bodenhausen et al., 2014; Dees et al., 2015; Gomba, Chidamba & Korsten, 2017; Jo et al., 2015; Rastogi et al., 2012; Truchado et al., 2017). Core microorganisms are not necessarily related to growth promoting pathways, but they somehow modulate the host to allow other microbes to associate with them (Toju et al., 2018). These commonly found members of the phyllosphere are interrelated both, ecologically and evolutionarily, with the host, representing the non-transient species inhabiting this environment (Kembel & Mueller, 2014).

At family level, we could identify a quite similar presence in both systems, such as *Methylobacteriaceae* (phylum: *Proteobacteria*) in both CO and EC, which can be representing the predominance of *Methylobacterium*, a stable and abundant taxon in the phyllosphere community, where its interaction with the host promotes several benefits (Table 3). Classified as methylotrophic bacteria, these microorganisms are capable of using plant-derived methanol (CH_3_OH) or methane (CH_4_) as sole source of carbon and energy, inducing plant immune defence and protection against UV radiation (Del Rocío et al., 2017; Senthilkumar & Krishnamoorthy, 2017). Metaproteogenomic analyses of several plant hosts identified the presence of proteins related to methanol used as carbon and energy source (Thapa & Prasanna, 2018). Therefore, the association of this genus with leaf surfaces can overcome an epiphytic relation to become a symbiotic interaction. Indeed, microbial composition correlates with the plant’s physiology in several cultures, in which a higher diversity can provide several benefits to the host. Moreover, as being the most representative microorganism on foliar surfaces, bacteria can often contribute to some metabolic traits that affect the host’s phenotype, and consequently promote a healthy development (Thapa & Prasanna, 2018). Because of the leaf surface conditions, such as high exposition to the sun and low nutrient availability, the phyllosphere tends to restrain its colonization to species that have metabolic strategies to comply with the host’s taxonomical and functional traits. Laforest-Lapointe, Messier & Kembel (2016) led some intra-individual studies on variations of the bacterial community structure, where the diversity shows to have a significant influence on host species in natural forest. Therefore, at this taxonomic level, these groups seem to be characteristic of the phyllosphere region, independently of the parameter evaluated, and the farming systems in our experiment did not interfere with the stable resident bacterial community.

Indeed, microbial composition correlates with the plant’s physiology in several crop plants, in which a higher diversity can provide several benefits to the host. Moreover, as being the most representative type of microorganism on foliar surfaces, bacteria can often contribute to some metabolic traits that affect the host’s phenotype, and consequently promote a healthy development (Thapa & Prasanna, 2018). We found evidence in the literature that correlates this beneficial interaction of host plants and microorganisms with increased productivity (Andreote & De CássiaPereira e Silva, 2017; Laforest-Lapointe et al., 2017), pointing out that management may have a contrasting contribution to the phyllosphere metabolism.

To identify the forces that drive the patterns of prevalence and abundance of microbial communities has been a challenge for phyllosphere studies. Our sequencing analysis provided an initial insight, where it is possible to verify divergence in microbial diversity between areas with differential management, as we had hypothesized. Further kinds of diversity investigations reinforced those results, demonstrating an evident distinction between bacterial community structures. Microbial richness in EC and CO did not show to be statistically different from each other, however diversity indices and contribution analysis demonstrated dissimilarities in community structure and, possibly, in dynamics as well (Table 2). Contributing analyses (Table 3) demonstrate that the main bacterial families to reflect the dissimilarity between areas are significantly derived from EC samples. Relative abundance of the most significant families is highly distributed among ecological samples, presuming that there is a better contribution for phyllosphere dynamics in this environment (Table 3). Plant traits can be improved by bacterial community composition as ecological strategies of the host related to its products, as we discussed for Methylobacteriaceae, but also for Acetobacteriaceae and Sphingomonadaceae (Kembel & Mueller, 2014; Smets et al., 2020). For instance, the significantly higher presence of Rhizobiales in the EC group could perhaps indicate an important symbiotic relationship with plant growth promoting bacteria, since Rhizobiales frequently act as such in some agricultural environments (Copeland et al., 2015; Peñuelas et al., 2012).

Thus, our sequencing results seem to indicate a favoring of microbial diversity in ecological farming, allowing more assorted groups from those samples. Ecological agriculture derives from the idea of trading the conventional techniques applied in the field for more sustainable alternatives, in order to find a balance among crop productivity and ecosystem functioning, while considering their impact on socioeconomic issues as well (Altieri, 2013; Ponisio et al., 2014). Furthermore, a conscious and well-designed transition to ecological agriculture can even promote long-term environmental conservation and restoration (Krauss, Gallenberger & Steffan-Dewenter, 2011). These environmental-friendly advantages endorsed by an ecological management can be responsible for the maintenance of greater biodiversity, with a positive effect on crop yield and, therefore, it can improve quality, *fitness* and even productivity of the plant cultivar (Andreote, Gumiere & Durrer, 2014; Andreote & De CássiaPereira e Silva, 2017).

Endorsing our hypothesis, PCoA data reflect a phylogeny-based assessment of bacterial community composition and, since this is based on weighted UniFrac, it still provides information about its structure (Fig. 3). β-diversity showed a believable influence of agricultural management on the composition of the bacterial community structure, suggesting it to be one of its drivers. We recognize that a greater number of replicates could reinforce our suggestion on microbial drivers, since this result is evidenced substantially in statistics, although the influence of crop management on differential microbial structures interacting with plants are also quite well documented. Thus our conclusions are easily corroborated by many reports that validate this occurrence in agro ecology (Toju et al., 2018). Perazzolli et al. (2014) demonstrated a partial effect of biological and chemical treatments on bacterial and fungal distribution on grapevines, where the pesticides were responsible for the modification of microbial communities as they changed the proportion of residents and pathogens. This imbalance between microorganisms could be a consequence of the broad-spectrum effect of most of the pesticides and other synthetic chemicals used in conventional agriculture. Inputs from either conventional or ecological management can promote divergences between leaf surfaces, allowing for the colonization and growth of different complex microbial communities (Ottesen et al., 2009). There is a correlation between microbial diversity and resistance to competitors, where the more diverse microbial community occupies all the available niches, decreasing the opportunities for invasive microorganisms (Eisenhauer et al., 2013; Van Elsas et al., 2012). This correlation can be applied to our microenvironment on the leaf surface, in which the rate of pathogens would decrease on the plant with a more stable and complex residential microbial community inhabiting a tissue that is very susceptible to infection.

After understanding the microbial community assemblage on the divergent phyllosphere, we quantified bacterial communities over time in order to observe eventual temporal differences. These data suggest possible effects of contrasting approaches in crop management, since we identified a relevant higher abundance of bacteria in EC during drought, suiting a tendency for the ecological treatment accomplishing more sustainable methodologies in the field. There also was a significant loss of abundance from EC across time, which may be related to the agricultural inputs.

It is important to point out that, in this year, during the rainy season, there was an unusually high attack of the “black spot” disease in both citrus areas, resulting in a not-predefined application of a copper-based fungicide that surpassed the established concentration limit (Table 1). Copper (Cu) is an essential micronutrient commonly used in traditional agriculture as a plant-sanitary treatment against microbial diseases due to its inhibitory potential on microbial growth (Datnoff & Elmer, 2018; Li et al., 2014). Conversely, in ecological agriculture, there is quite a limitation in the concentration of Cu used of not more than 6 kg ha^−1^ per year (Ministry of Agriculture, Livestock and Food Supply (MAPA), 2011). Indeed, this premise was followed in our experimental area during almost the whole experiment, until this infestation appeared at the very end.

Homma (2017), reported agricultural productivity of the same citrus experimental area but concerning the soil microbiota structure. His results showed a streight correlation between greater foliar Cu application and lower fruit productivity as one of the most significant variables to promote dissimilarity in fruit productivity and microbial diversity between conventional and ecological management during 2015/2016. This, as a matter of fact, was our major incentive to evaluate copper retention on foliar leaf tissue of CO and EC areas, in order to elucidate the possible causes. We were able to demonstrate Cu-accumulation on leaf tissue demonstrated by µ-XRF mapping, allowing us to follow Cu distribution during a 15-day period (Fig. 4; Fig. S1), in which CO leaves showed a lower Cu accumulation than EC management.

Such reasoning may also be associated to the reason why Homma’s results showed the same lower result of bacterial abundance in CO than in EC during the dry climate. Even though the micro-biocide effectiveness of Cu-based products is well-described in the literature (Ottesen et al., 2015; Richard et al., 2017), its ecotoxicological profile presents an important risk for its intense use (Dagostin et al., 2011), which normally is not considered in traditional agriculture. Copper is classified as a conditionally mobile element, which means that its absorption and transportation through the phloem is quite slow; therefore the rain scarcity at the sampling period contributed to Cu accumulation on the leaf tissue (Fernández, Sotiropoulos & Brown, 2015; Srivastava, 2012). In our investigation, we may seemingly correlate the loss of bacterial abundance to increased copper application because of black spot in EC samples, when we found a great decrease in the bacterial quantity, whose resident community probably was affected, as this community is not adapted to higher Cu concentrations. On the other hand, no significant change was identified in Cu abundance in CO, where there is no application limit for Cu. Martins et al. (2013, 2014) demonstrated the effect of Cu-based products on bacterial and fungal communities in grape phyllosphere. The higher concentrations used in the areas under conventional agricultural methods reduced the microbial communities in diversity and abundance, indicating a negative effect of the excessive use of Cu-derived products. Therefore, the correlation between bacterial decrease and copper accumulation in foliar tissue seems to be quite feasible.

For all these reasons it seems very probable that our assumptions are adequate, and the agricultural management system is an important driver of the phyllosphere microbial structure. We understand that the micro-XRF mapping was somewhat limited, however also suitable according to previous information about Cu retention and interference in foliar dynamics, regarding the microbial community. In the future, we indicate that pesticide dosage, frequency of application and mechanism of action are among the factors to be investigated in relation to the promotion of different consequences to the crop (Perazzolli et al., 2014). Further studies should attempt to investigate if copper usage can also interfere with the fungal community composition.

## Conclusions

Crop-independent methods show the significant effects of agricultural management, presenting the differences in bacterial diversity depending on the practices applied. The importance of the understanding of these effects caused by different management systems should be used in order to benefit food production and optimize practices for a more sustainable agro ecosystem.

## Supplemental Information

10.7717/peerj.9152/supp-1 Supplemental Information 1 Duplicates of Microchemical maps obtained by micro-XRF for Cu in the surface of *Citrus* sp. leaves. Duplicates of maps obtained by micro-XRF for Cu in the surface of *Citrus* sp. leaves conventional (A and B) and ecological management (C and D) on copper-based products application day (on the left) and 15 days-after (on the right). The Rh K-alpha Compton peak was used for correcting the maps.

10.7717/peerj.9152/supp-2 Supplemental Information 2 Raw data for 16S rRNA NGS.

## Additional Information and Declarations

### Data Availability

The following information was supplied regarding data availability: The data is available at the MG-RAST metagenomic analysis server: Citrus phyllosphere 16SrRNA (mgp88102). MGRAST-ID: mgm4832466.3; mgm4832467.3; mgm4832468.3; mgm4832469.3; mgm4832470.3; mgm4832471.3. The raw data is also available as a Supplemental File. This should be linked to “Citrus phyllosphere 16SrRNA (mgp88102)”: https://www.mg-rast.org/mgmain.html?mgpage=project&project=mgp88102.
