# Supplementary figures and images for: Phyllosphere bacterial assembly in citrus crop under conventional and ecological management
Source: PeerJ. 2020 Jun 2;8:e9152. doi: 10.7717/peerj.9152 (PMC7274167; doi:10.7717/peerj.9152)

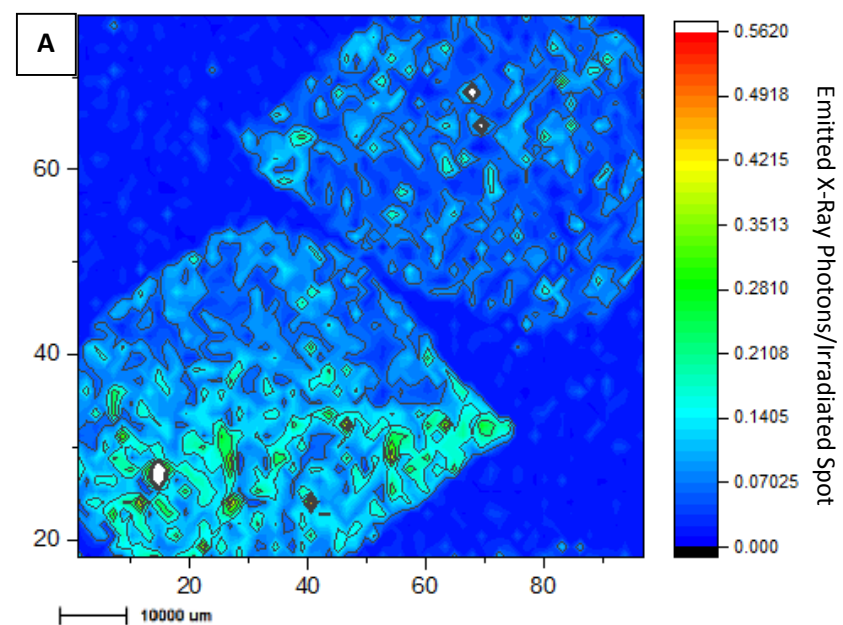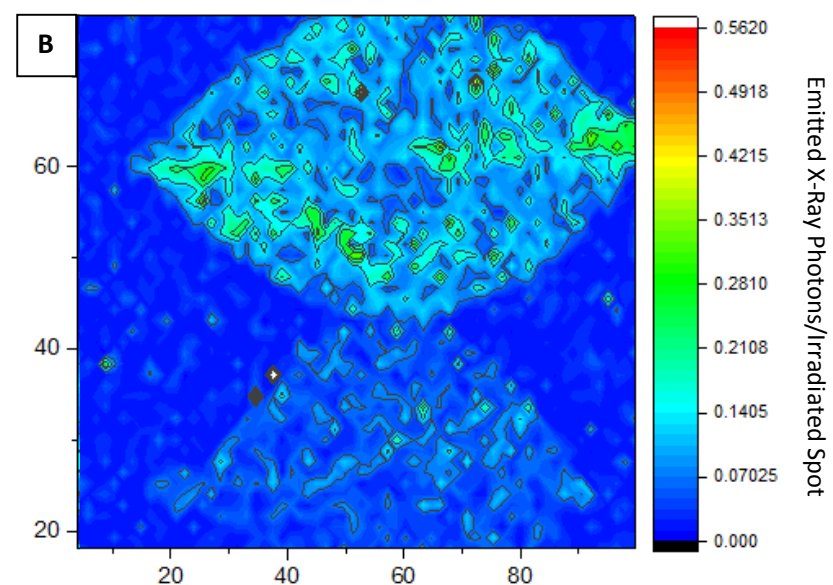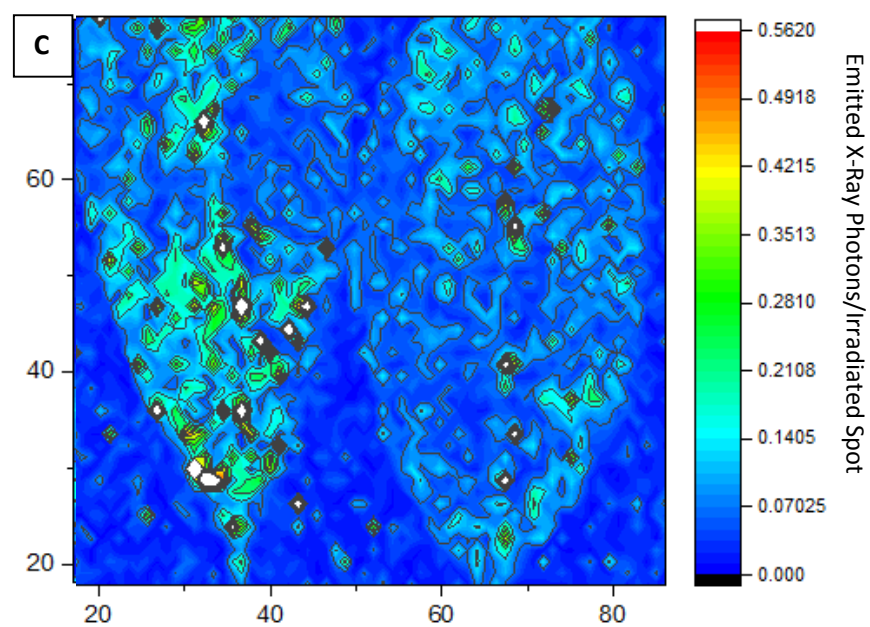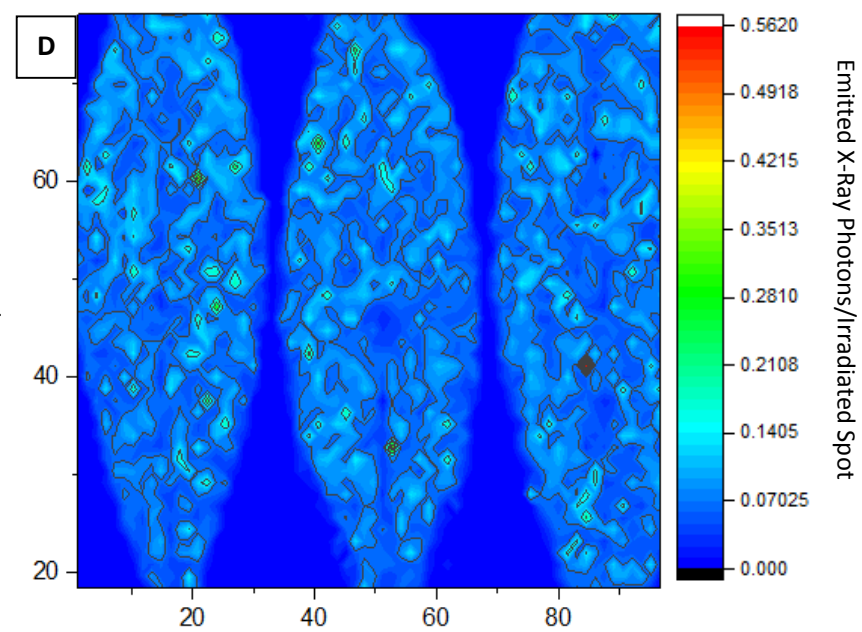

Supplement: Supplemental Information 1 — Duplicates of maps obtained by micro-XRF for Cu in the surface of Citrus sp. leaves conventional (A and B) and ecological management (C and D) on copper-based products application day (on the left) and 15 days-after (on the right). The Rh K-alpha Compton peak was used for correcting the maps. [file peerj-08-9152-s001.pdf]
